# Efficient production of a mature and functional gamma secretase protease

**DOI:** 10.1038/s41598-018-30788-w

**Published:** 2018-08-27

**Authors:** Imran Khan, Sudarsan Krishnaswamy, Miheer Sabale, David Groth, Linda Wijaya, Michael Morici, Imre Berger, Christiane Schaffitzel, Paul E. Fraser, Ralph N. Martins, Giuseppe Verdile

**Affiliations:** 1 School of Biomedical Sciences, Faculty of Health Sciences, Curtin Health Innovation Research Institute, Curtin University, Bentley, Western Australia Australia; 2 School of Psychiatry and Clinical Neurosciences, University of Western Australia, Crawley, Western Australia Australia; 3 Centre of Excellence for Alzheimer’s Disease Research & Care, School of Medical Sciences, Edith Cowan University, Joondalup, Western Australia Australia; 4 European Molecular Biology Laboratories, Grenoble, France; 5 School of Biochemistry, University of Bristol, Bristol, UK; 6 Tanz Centre for Research in Neurodegenerative Diseases and Department of Medical Biophysics, Krembil Discovery Tower, University of Toronto, Toronto, Ontario Canada; 7 Department of Biomedical Sciences, Faculty of Medicine and Health Sciences, Macquarie University, Sydney, New South Wales Australia; 8 School of Psychology and Exercise Sciences, Murdoch University, Murdoch, Western Australia Australia

## Abstract

Baculoviral protein expression in insect cells has been previously used to generate large quantities of a protein of interest for subsequent use in biochemical and structural analyses. The MultiBac baculovirus protein expression system has enabled, the use of a single baculovirus to reconstitute a protein complex of interest, resulting in a larger protein yield. Using this system, we aimed to reconstruct the gamma (γ)-secretase complex, a multiprotein enzyme complex essential for the production of amyloid-β (Aβ) protein. A MultiBac vector containing all components of the γ-secretase complex was generated and expression was observed for all components. The complex was active in processing APP and Notch derived γ-secretase substrates and proteolysis could be inhibited with γ-secretase inhibitors, confirming specificity of the recombinant γ-secretase enzyme. Finally, affinity purification was used to purify an active recombinant γ-secretase complex. In this study we demonstrated that the MultiBac protein expression system can be used to generate an active γ-secretase complex and provides a new tool to study γ-secretase enzyme and its variants.

## Introduction

Multi-protein complexes have vital roles in many cellular functions^1,2^. One such complex is the transmembrane γ-secretase enzyme that is responsible for proteolytically cleaving numerous Type I transmembrane proteins^2,3^. For example, Amyloid Precursor Protein (APP) is proteolytically cleaved by γ-secretase to generate various Aβ peptides, some of which have been shown to accumulate in Alzheimer’s disease (AD) brains^4^. Amyloidogenic processing of APP is initiated by β-APP cleaving enzyme-1 (BACE1) that cleaves the ectodomain of APP to generate a membrane embedded APP C-terminal fragment (C99). This APP-C99 fragment is subsequently processed by γ-secretase to generate multiple Aβ peptides and the APP intracellular domain (AICD)^5^. The generation and accumulation of longer Aβ peptides (e.g. Aβ42) plays a key role in the events that lead to neurodegeneration in the AD brain^6^. Therefore, γ-secretase is a logical target for the development of inhibitors/modulators aimed at lowering Aβ production. However, the complexity of the enzyme and its ability to process many different substrates have hindered targeted, therapeutic development efforts. Undesirable off-target effects related to disruption of Notch signalling are observed in animal and human trials^7–10^, therefore highlighting a need for a better understanding of γ-secretase’s structure and function.

The enzyme consists of a combination of multi-pass transmembrane proteins, Presenilin (PS1 or PS2), Nicastrin (NCT), Anterior Pharynx Homologue 1 (APH1a [long/short isoform] and APH1b in human and in addition Aph1c in mice) and Presenilin Enhancer 2 (PEN-2). Although high-resolution structural studies^11–15^ have provided an insight into the process of γ-secretase assembly and activity, additional information is still required. An understanding of the flexible domains of γ-secretase that are responsible for recognition, selection, sorting and shuttling substrate to the active site^16,17^ is required. Sampling of different dynamic conformations of γ-secretase^11,13,14^, will provide us with an insight into the molecular mechanism^18^ essential for substrate specific drug developmental strategies^17^. Furthermore, an elucidation of the substrate-γ-secretase interaction will assist in developing disease specific therapeutics that target APP processing in Alzheimer’s disease and Notch processing in certain cancers^17^, cerebral autosomal dominant arteriopathy with subcortical infarcts and leukoencephalopathy (CADASIL)^19^ and acne inversa^20^. In addition, as the γ-secretase enzyme is involved in a large number of other physiological processes by virtue of its large substrate cohort^2^, more detailed information pertaining to this enzyme will enable us to better understand its molecular mechanisms in disease and physiology.

Functional, structural and molecular insights into the γ-secretase enzyme can be obtained through reconstructing the enzyme using an appropriate expression system and generating large amounts of pure, active γ-secretase complex. Moreover, a robust system allows for generation of various combinations of different γ-secretase component isoforms/homologues. In line with the aforementioned requirements, we investigated the use of a multi protein baculoviral expression system to reconstitute an active γ-secretase enzyme complex. Baculovirus mediated recombinant protein expression in insect cells is a suitable eukaryotic system, and is especially useful in generating large amounts of proteins and protein complexes^21^. The flexible baculoviral capsid allows packaging of large heterologous genes (>20 Kb) and recombinant protein expression can range up to 50% of insect cell proteins^21^. Thus, in comparison to yeast and mammalian systems, higher expression of large proteins/protein complexes is achieved. In addition, yeast and mammalian cells express endogenous γ-secretase and other proteases with γ-secretase like activity and γ-secretase binding proteins^22^. These endogenous proteins could potentially influence recombinant γ-secretase expression, assembly, stoichiometry and activity^23,24^. Additional benefits of the baculovirus expression system are in its ease of use, consistency in recombinant protein expression and adaptability to large bio-reactor scale expression setups. These characteristics make baculoviral expression an appealing system to reconstitute γ-secretase enzyme complex.

Previously, baculoviral expression systems have been used to express recombinant γ-secretase, either by expressing individual components^25,26^ or the whole γ-secretase complex by co-infecting insect cells with multiple baculoviruses each containing a single core-component protein^25,27^. However, co-infection with multiple baculoviruses to reconstitute γ-secretase is technically challenging and laborious. Co-infection augments baculovirus mediated impairment of cellular post-translational machinery, protein degradation and results in poor recovery of recombinant protein^28^. Deleterious effects associated with co-infection makes stoichiometric expression of γ-secretase components rather difficult^29^. Therefore, baculoviral co-infection approaches do not seem amenable to expressing large quantities of active multiprotein complexes like γ-secretase. In contrast, the MultiBac baculovirus expression system, utilises a single plasmid (pFBDM) to drive near stoichiometric expression of the complex proteins, thus circumventing limitations associated with traditional baculoviral expression systems^30,31^. Additionally, MultiBac employs a recombinant baculovirus DNA with disabled viral cathepsin and chitinase (cysteine protease) genes^30^ that are involved in viral mediated cellular liquefaction^32,33^. This enables better compartmentalization and reduced viral protease mediated degradation, of recombinant proteins^30^. The multicistronic vector, pFBDM, facilitates the generation of a single recombinant baculovirus containing multiple expression cassettes with identical or similar efficacy promoters (baculoviral promoters of highly expressed polyhedron ‘polH’ and P10 proteins). This approach achieves uniform concurrent expression of multiple proteins^30,31^. In addition, the combinatorial gene assembly method employed in MultiBac, is particularly useful to generate variations of a γ-secretase enzyme complex (such as different subunits and isoforms, mutants etc.) and investigate potential protein modulators of γ-secretase activity (i.e. CD147, TMP21, Rab21^3,34–36^).

The versatility and flexibility of the MultiBac expression system is demonstrable in its successful application to the production of large protein complexes (including polytopic transmembrane protein complexes), the development of Virus-like particle (VLP) based vaccines and gene therapy vectors^37–40^. The MultiBac expression system has been used to reconstitute a 14 subunit complex, transcription factor IID^41^ and a bacterial translocon protein complex^42,43^. In this study, we use the MultiBac baculoviral system to reconstitute an active, multi-subunit transmembrane γ-secretase enzyme complex in insect cells. We also expressed an Autosomal Dominant Alzheimer’s Disease (ADAD) related PS1 mutant- missing exon 9 (PS1Δ9). The PS1 mutation-PS1(Δ9) is of particular interest in understanding the molecular mechanisms of γ-secretase mediated proteolysis. PS1(Δ9) does not need endoproteolytic activation (lacks exon 9 wherein lies the endoproteolytic site), to process γ-secretase substrates^26,44,45^.

## Results

### Reconstitution of γ-secretase using MultiBac baculovirus

**Figure 1:**
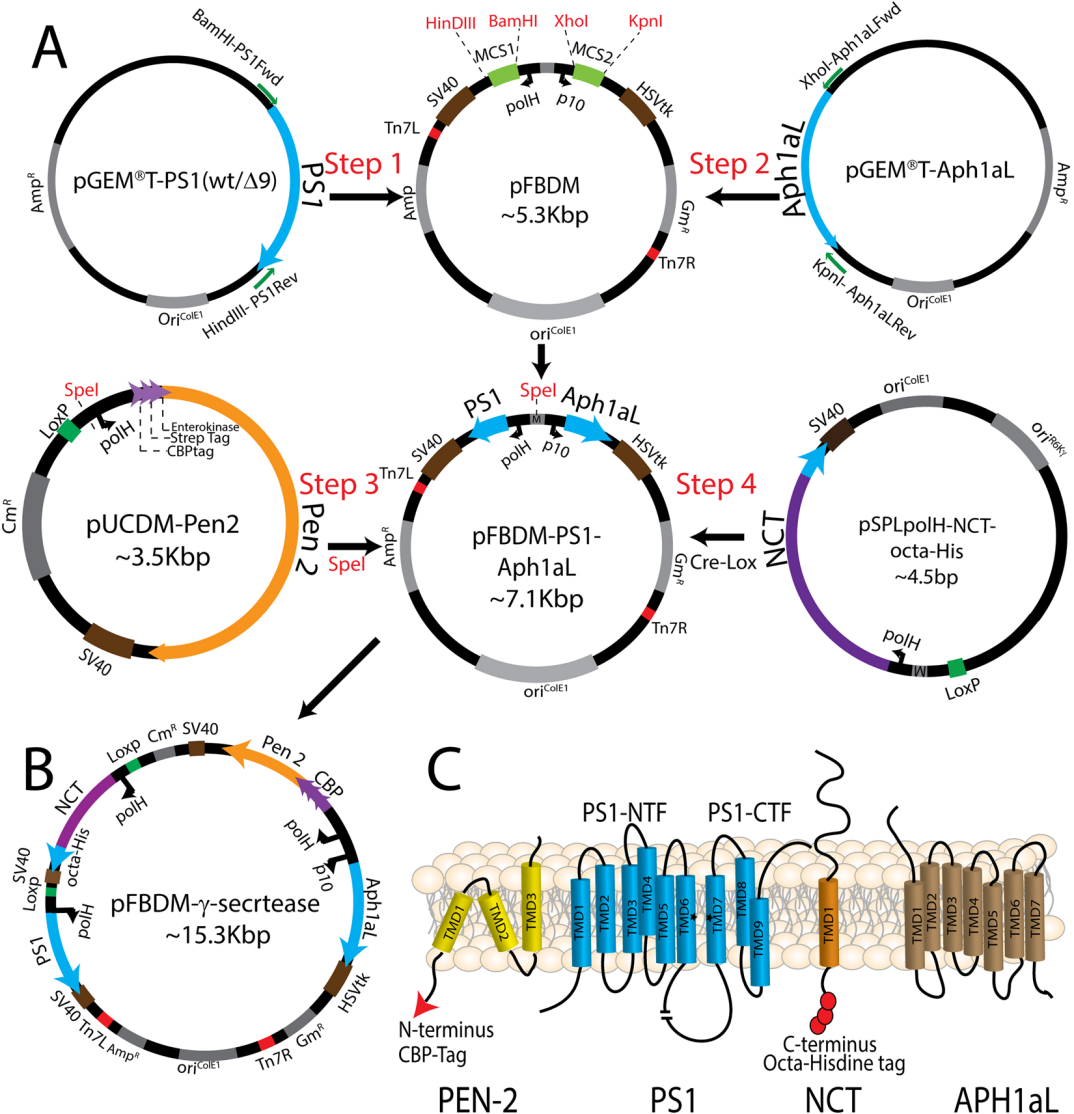
Cloning Strategy to incorporate cDNA encoding γ-secretase components into the MultiBac pFBDM plasmid. PS1 and APH1aL cDNAs were cloned from pGEM®T vectors. PEN-2 and Nicastrin DNA constructs were cloned into the pUCS and pSPL vectors respectively. The polH and p10 annotations indicate the promoters of the respective baculoviral genes (**A**) Step 1: PS1 was cloned at multiple cloning site 1 (MCS1) of the pFBDM plasmid to generate pFBDM-PS1. Step 2: APH1aL was cloned into pFBDM-PS1 at the MCS2 to generate pFBDM-PS1-APH1aL. Step 3: PEN-2 cDNA construct was cloned at the SpeI restriction site on pFBDM-PS1-APH1aL. Step 4: The pSPLpolH-NCT-octa-his was cloned into pFBDM-PS1-Aph1aL-Pen-2 generated in step 3 by Cre-lox recombination to generate pFBDM-γ-secretase (**B**) A map of the pFBDM-γ-secretase used to generate recombinant baculoviruses (**C**) A schematic of recombinant γ-secretase enzyme expressed in insect cells showing protein purification tags; PEN-2 tagged with Calmodulin Binding Protein (CBP) at the N-terminus (red arrowhead) and NCT expressed with an “octa-histidine tag” at the C-terminus (red circles).

**Figure 2:**
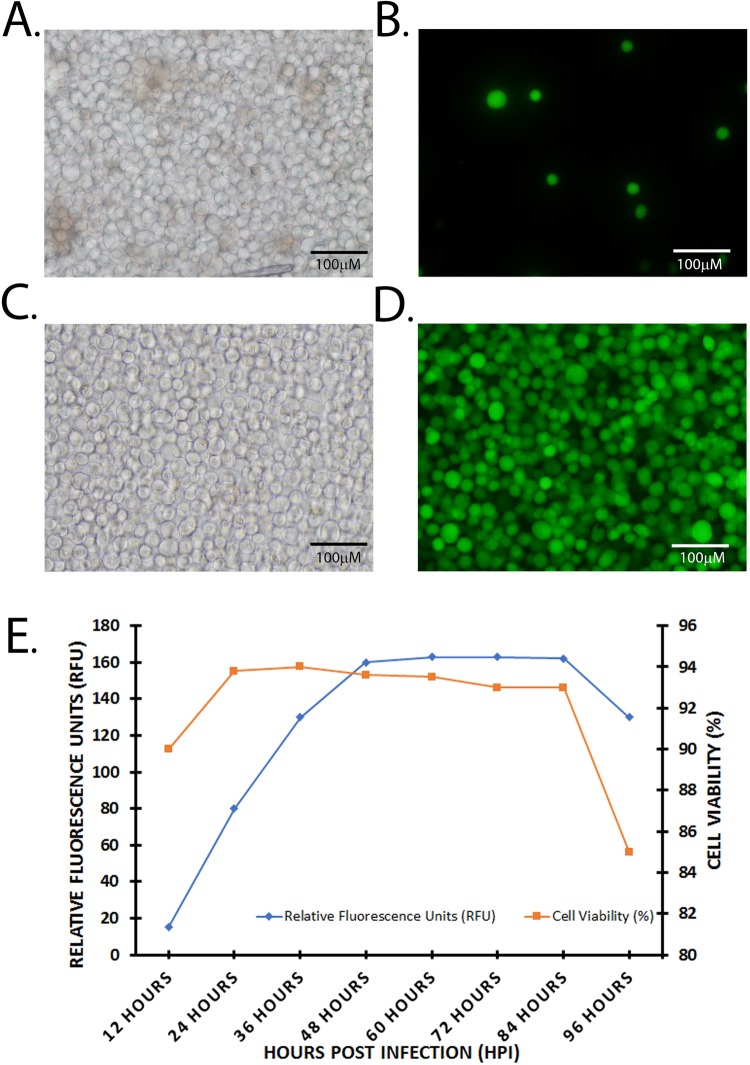
Monitoring cell viability and YFP expression. Representative pictures of baculovirus infected Sf9 cells viewed using bright field (**A,C**) or fluorescent microscopy (**B,D**). Sf9 cells infected with empty baculovirus EmBacY (**A,B**) or those infected with the recombinant baculovirus expressing PS1(wt)-γ-secretase (**C,D**). (**E**) V2 recombinant baculoviruses were used to infect Sf9 cells (1 × 10^6^ cells/ml; ~90% viability) at MOI of 1 PFU. Every 12 hours post infection (HPI) 1 × 10^6^ cells were harvested, and YFP expression was monitored by using a plate reader at ~520 nm against a PBS blank. Relative fluorescence units were plotted against time. Magnification 20x, Scale Bar 100 μm.

**Figure 3:**
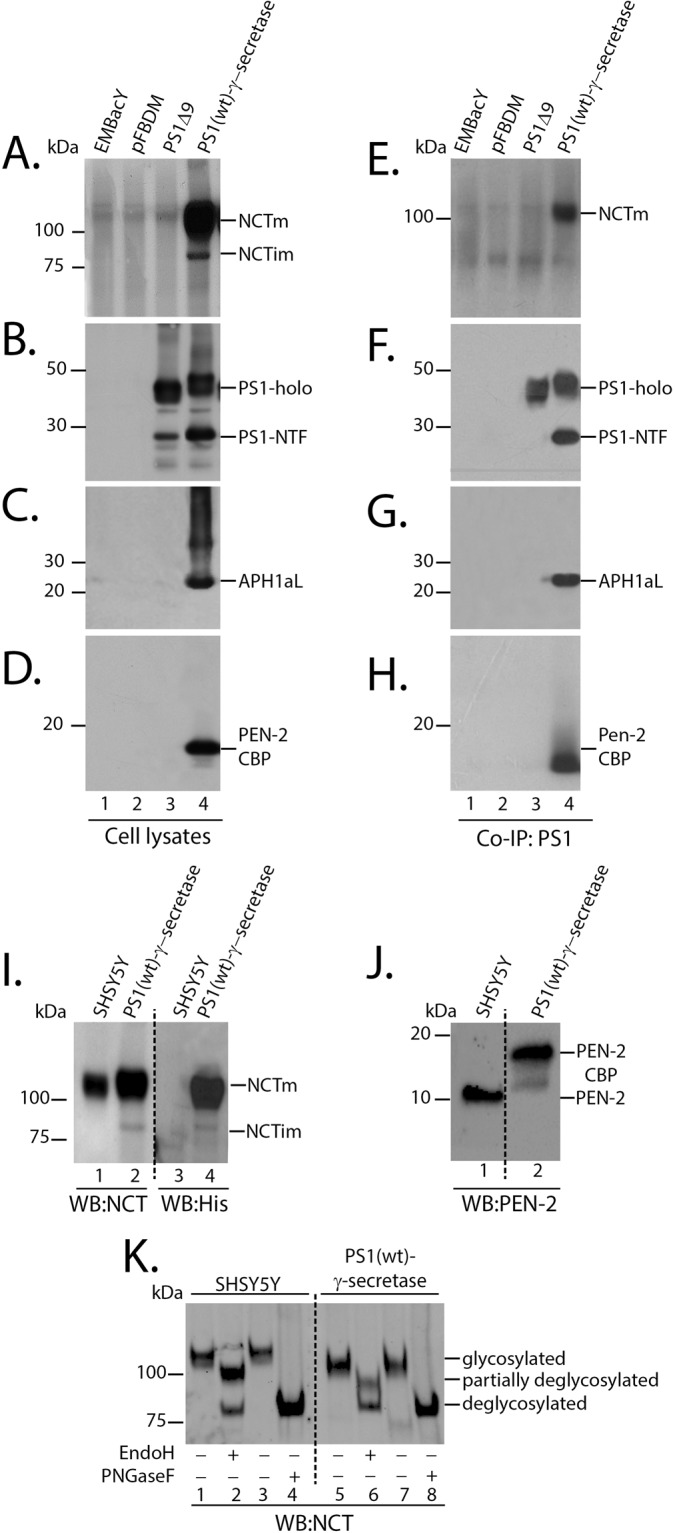
The γ-secretase components are expressed and interact with PS1. Expression of all γ-secretase enzyme components was observed in whole cell lysates of insect cells infected with recombinant baculovirus expressing components was observed in whole cell lysates of insect cells infected with recombinant baculovirus expressing PS1(wt)-γ-secretase (**A–D**, lane 4). No such expression was observed in cells infected with control baculovirus EmBacY (**A–D**, lane 1) or recombinant baculovirus with control pFBDM plasmid (**A–D**, lane 2). Both PS1 holoprotein and PS1-NTF (antibody Ab14) were observed in insect cells infected with recombinant baculoviruses PS1(wt)-γ-secretase (**B**, lane 4); indicating successful endoproteolysis of PS1. Robust expression of mature (**A** lane 4, ~110 KDa) and minimal expression of immature (**A** lane 4; ~80 Kda) Nicastrin was observed. (**E–H**) Immunoprecipitation using PS1 Ab14 antibody was performed on γ-secretase expressing insect cell membranes. All four components of γ-secretase enzyme co-immunoprecipitated with PS1 antibody, indicating interaction with PS1. Only mature NCT (**E** lane 4; ~110 kDa) was observed to interact with PS1. (**F**) PS1 antibody NT1 was used to detect immunoprecipitated PS1. (**I**) Western immunoblotting of SHSY5Y and PS1(wt)-γ-secretase expressing insect cells with NCT antibody (lanes 1 and 2) and penta His antibody (lanes 3 and 4) detected “octa-His” tag on PS1(wt)-γ-secretase only (lane 4). (**J**) Western immunoblotting of SHSY5Y and PS1(wt)-γ-secretase expressing insect cells with PEN-2 antibody detected PEN-2-CBP at a higher molecular weight (lane 2; ~15 kDa) due to CBP tag compared to endogenous PEN-2 (lane 1; ~10 kDa). (**K**) Deglycosylation of Nicastrin from SHSY5Y and PS1(wt)-γ-secretase expressing insect cells with Endo-H (lanes 2 and 6) and PNGase-F (lanes 4 and 8). Nicastrin expressed in insect cells showed partial resistance to Endo-H (lane 6), similar to SHSY5Y endogenous Nicastrin (lane 2), indicating complex glycosylation in insect cells. Insect cell Nicastrin was highly susceptible to PNGase-F mediated deglycosylation (lane 8), similar to SHSY5Y endogenous Nicastrin (lane 4). No such deglycosylation events occurred in control experiments setup without Endo-H (lanes 1 and 5) and PNGase-F (lanes 3 and 7).

The cloning strategy used to insert the γ-secretase components into the pFBDM plasmid is outlined in Fig. 1A,B, with included protein purification tags are shown in Fig. 1C (octa-Histidine at the C-terminus of NCT and Calmodulin Binding Protein at N-terminus of PEN-2) and detailed in Materials and Methods. The final recombinant plasmid, termed pFBDM-γ-secretase, underwent restriction digestion analysis using HinDIII (Fig. S1). DNA sequencing was used to confirm that the component gene inserts were incorporated into the pFBDM in the correct orientation. DH10EmBacY *E. coli*, was transformed with pFBDM constructs to generate recombinant EmBacY bacmid. The EMBacY bacmid contains a YFP reporter gene to monitor viral performance and expression of heterologous proteins in insect cells^30^. Sf9 cells were transfected with recombinant EmBacY bacmid extracted from DH10EMBacY *E. coli* to generate the initial baculoviruses (V0). Subsequently V1 and V2 were generated and baculoviral titre was determined on V2. A 50 ml suspension of Sf9 cells (1 × 10^6^ cells/ml; >90% viability), was infected with baculovirus (V2) at multiplicity of infection (MOI) of 1 PFU (plaque forming unit/cell). Robust YFP expression was observed at 48 hours post infection (HPI) (Fig. 2D compared to Fig. 2B- control EmBacY baculovirus; Fig. 2A,C are respective bright field images). The YFP expression plateaued over the next 48–84HPI and then declined between 84–96HPI, concomitant with a reduction in cell viability to ~85% (Fig. 2E). A similar expression/viability profile was obtained for the PS1Δ9 baculoviruses (data not shown). The cells were harvested and whole cell lysates were prepared for the detection of the components of γ-secretase by immunoblotting (Fig. 3A–D) or cell membranes were prepared for co-immunoprecipitation experiments to analyse the interaction between the expressed components of γ-secretase (Fig. 3E–H).

Immunoblotting of cell lysates from PS1(wt)-γ-secretase infected cells, showed expression of both PS1-holoprotein and PS1 N-terminal fragment (PS1-NTF) (~45 KDa and 28 kDa, respectively; Fig. 3B, lane 4), indicating that the PS1 holoprotein undergoes endoproteolytic activation in insect cells^46–48^. Robust expression of Nicastrin (Fig. 3A, lane 4), APH1aL (Fig. 3C, lane 4) and PEN-2 (Fig. 3D, lane 4) was also observed. All components were shown to co-immunoprecipitate with PS1 (Fig. 3E–H, lane 4), indicating interaction between the components^29^. In addition, only the mature/glycosylated form of Nicastrin (Fig. 3E, lane 4; ~110 KDa) co-immunoprecipitated with PS1, consistent with an intact functional complex^49^. Immunoblotting using the penta-his antibody detected the “octa-His” tag from insect cell expressing Nicastrin (Fig. 3I; lane 4) and not endogenous Nicastrin from human neuroblastoma SHSY5Y cells (Fig. 3I; lane 3), while the Nicastrin antibody detected NCT in both the SHSY5Y (Fig. 3I; lane 1) and PS1(wt)-γ-secretase expressing insect cells (Fig. 3I; lane 2). Immunoblotting using the PEN-2 antibody detected insect cell expressed PEN-2-CBP at a higher molecular weight (Fig. 3J; lane 2; ~15 kDa) compared to SHSY5Y endogenous PEN-2 (Fig. 3J; lane 1; ~10 kDa). In accordance with previous reports, the presence of these tags did not appear to alter the interaction of the components with PS1 or activity (see below)^50–53^. Deglycosylation of insect cell expressed and SHSY5Y endogenous NCT indicated similar partial resistance to Endoglycosidase-H (Endo-H) (Fig. 3K; lane 2 and lane 6), and high susceptibility to Peptide-N-glycosidase F (PNGase-F) mediated deglycosylation (Fig. 3K; lanes 4 and 8).

Immunoblotting of insect cell lysates expressing PS1(Δ9) showed expression of the PS1 holoprotein (Fig. 3B, lane 3). It was noted, however, that additional protein bands ranging from ~15–30 kDa were detected with the polyclonal PS1 N-terminal antibody, Ab14. The relevance of these additional protein bands is unclear, particularly following expression of the PS1(Δ9), which lacks exon 9 and thus cannot undergo endoproteolysis and thereby could possibly indicate nonspecific proteolysis. These lower molecular weight fragments, were absent following immunoprecipitation with Ab14 and immunoblotting with the monoclonal antibody NT1 (Fig. 3F, lane 3), indicating that they may represent degradation fragments/non-specific artifact.

### MultiBac reconstituted γ-secretase enzyme complex is active

**Figure 4:**
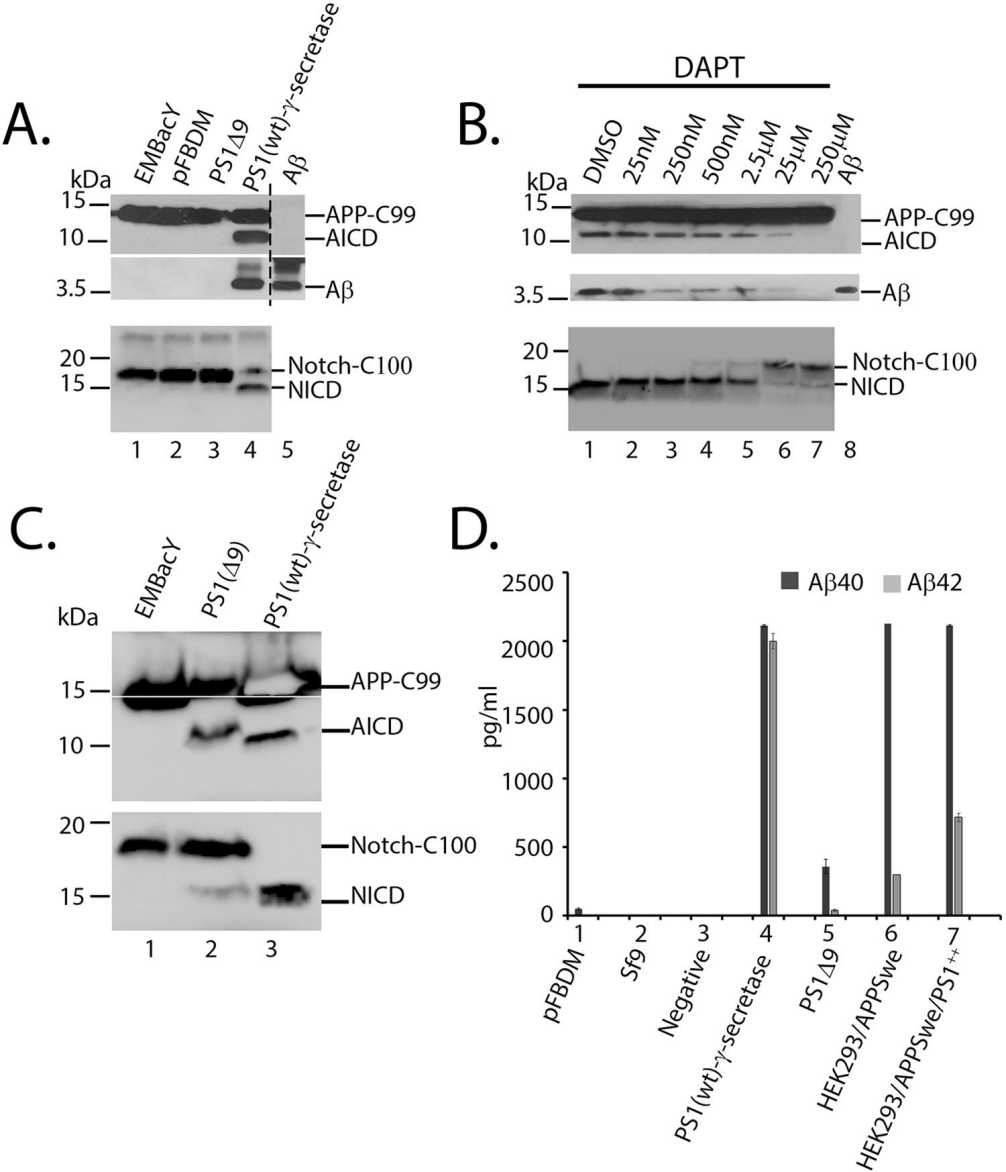
MultiBac γ-secretase shows activity in cleaving APP-C99 and Notch-C100. Cell free assay using insect cell membranes in 0.25% CHAPSO were incubated with APP-C99-Flag (A, and B, top and middle panels) or Notch-C100- Flag (A and B bottom panel) substrates. AICD and NICD were detected with anti-Flag antibody and Aβ with WO2 antibody. (**A**) PS1(wt)-γ-secretase lead to formation of AICD (lane 4 top panel), Aβ (lane 4 middle panel, run on the same gel) and NICD (lane 4 bottom panel). (**B**) The activity of PS1(wt)-γ-secretase could be inhibited by γ-secretase inhibitor DAPT in a dose dependant manner and prevented formation of AICD (top panel), Aβ (middle panel, run on the same gel) and NICD (bottom panel). (**C**) Cell free assay setup with PS1(Δ9) gave rise to AICD (lane 2 top panel) and NICD (lane 2 bottom panel) when used at 4 times the concentration of PS1(wt)-γ-secretase (lane 3). (**D**) Cell free assays were setup as above using cell membranes prepared from insect cells expressing PS1(wt)-γ-secretase or PS1(Δ9) or mammalian cells HEK293/APPSwe or HEK293/APPSwe/PS1++ cells. The cell free assay products were analysed by sandwich ELISA. Baculovirally reconstituted PS1(wt)-γ-secretase produced similar levels of Aβ40 when compared to mammalian HEK293 cells stably expressing APPSwe (HEK293/APPSwe) and HEK293/APPSwe cells transiently overexpressing PS1 (HEK293/APPSwe/PS1++). However, in comparison PS1(wt)-γ-secretase generated significantly larger amounts of Aβ42. PS1(Δ9) generated very little Aβ40 and Aβ42.

The activity of the recombinant PS1(wt)-γ-secretase and PS1(Δ9) was evaluated in cell free assays. PS1(Δ9) does not require other core γ -secretase components to process γ-secretase substrates^26,44,45^. Cell free assays were prepared with both APP-C99 or Notch-C100 substrates and the Aβ/AICD (APP intracellular domain) and NICD (Notch intracellular domain) proteolytic cleavage fragments were detected by immunoblotting^54,55^. AICD and NICD were detected in cell free assay reactions performed with PS1(wt)-γ-secretase containing insect cell membranes, while these products were absent in control cell free assays (Fig. 4A, top and bottom panels compare lanes 1 and 2, with lane 4). Immunoblotting using WO2 (5–8 amino acids of Aβ) detected a ~4 kDa protein that migrated at a similar size as synthetic Aβ peptide (Fig. 4A, middle panel, compare lane 4 with lane 5). ELISA was used to confirm/quantitate Aβ40 and Aβ42 generated in cell free assays (Fig. 4D). These results indicate that the reconsituted wild-type γ-secretase complex is actively processing the two substrates. A dose dependent reduction in the formation of the AICD/Aβ and NICD fragments following treatment with the γ-secretase inhibitor, DAPT (a highly potent γ-secretase inhibitor with similar efficacy in blocking APP and Notch processing in *in vitro* cell free assays^56^), also supported the PS1(wt)-γ-secretase activity (Fig. 4B).

In contrast, when the activity of PS1(Δ9) was examined in cell free assays, four times more protein was required to detect AICD and NICD fragments compared to PS1(wt)-γ-secretase (Fig. 4C). In addition, the quantity of Aβ generated, as determined by ELISA, in the PS1(Δ9) cell free assays was markedly reduced relative to the equivalent PS1(wt)-γ -secretase assays (Fig. 4D). As shown in previous studies, PS1(Δ9) also led to the production of significantly less Aβ42 compared to Aβ40^44,45^. These results indicate PS1(Δ9) is less efficient in processing γ-secretase substrates, consistent with previous studies^26,44,45,57–59^.

### Purification of PS1(wt)-γ-secretase

**Figure 5:**
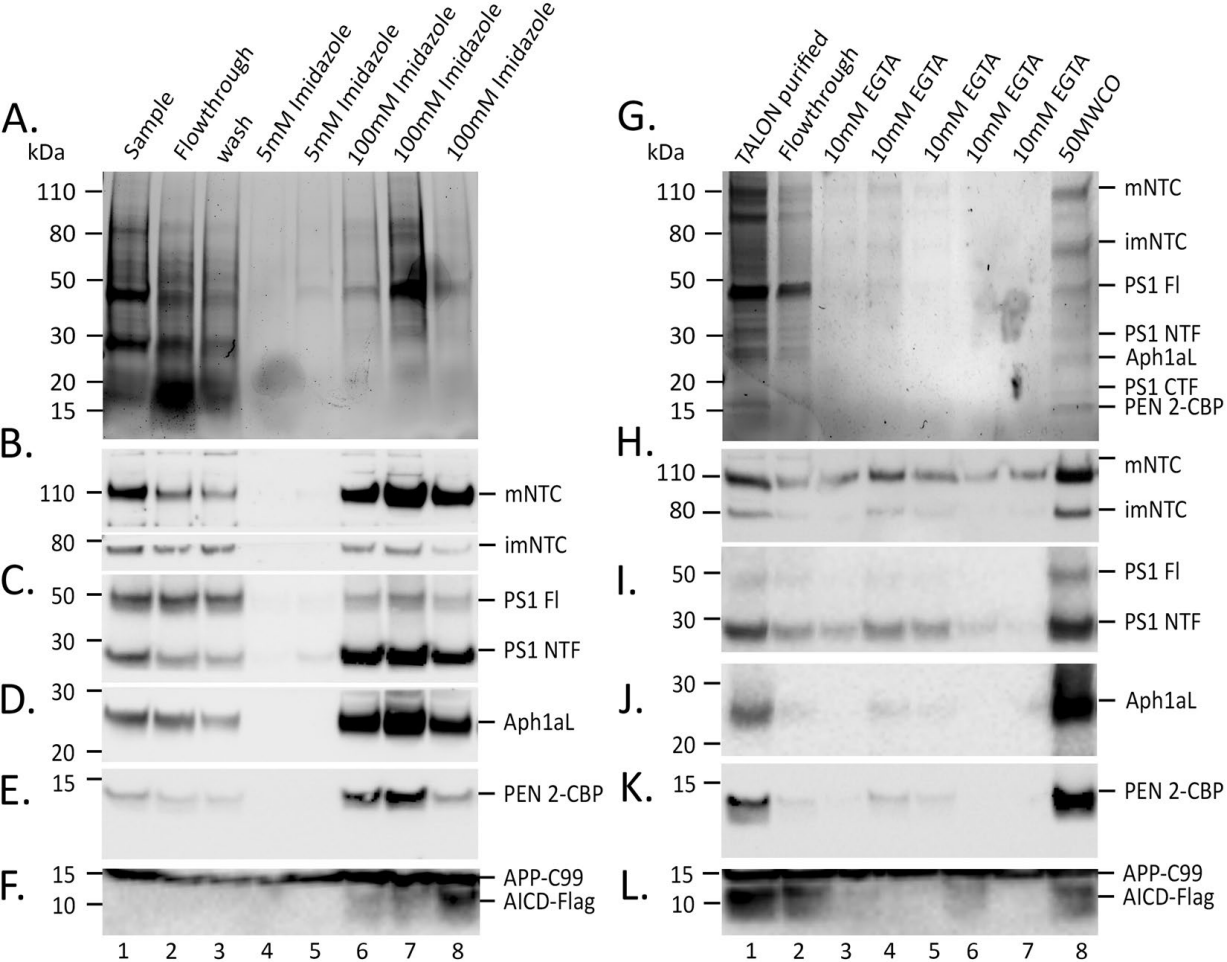
Purification of baculovirally reconstituted γ-secretase enzyme complex. Cell membranes of PS1(wt)-γ-secretase expressing insect cells were prepared in 1% CHAPSO and diluted to 0.08% CHAPSO and 0.08% Digitonin. (**A**) Coomassie stained SDS-PAGE of IMAC purified γ-secretase fractions. Membrane preparations (0.08% CHAPSO +0.08% digitonin in Hepes buffer; Buffer **B**) from baculovirally expressed γ-secretase following His-Tag purification. Lane 1, diluted membrane preparation sample; lane 2 Flow-through; lanes 3, wash fraction. Lanes 4–8, samples collected from column following elution with 5 mM Imidazole (lane 4 and 5) or 100 mM Imidazole (lane 6–8). All components were present in fractions eluted with 100 mM imidazole (**B**–**E**). (**F**) Cell free assay to assess activity of the fractions to cleave APP-C99 substrate and generate AICD. AICD was only observed with in the sample eluted with 100 mM imidazole (lanes 6–8). (**G–K**) IMAC purified 100 mM imidazole factions were pooled (lane 1) supplemented with 2 mM CaCl2 and 2 mM MgOAc and applied to Calmodulin resin (CaM) and eluted in 3 ml each of 10 mM EGTA (lanes 3–7) in 0.5% CHAPSO Tris-Buffer (Buffer **D**). Eluted fractions were pooled and concentrated with 50 kDA filter before SDS-PAGE (**G**) Coomassie stained SDS-PAGE of CaM purified γ-secretase components detected all γ-secretase components in 50 kDa filter concentrated sample (lane 8). (**H**–**K**) Immunoblotting detected γ-secretase components enriched in concentrated fraction (lane 8). (**L**) Cell free assay setup with CaM purification fractions and APP-C99 detected AICD fragment in starting TALON purified fraction (lane 1), CaM flowthrough (lane 2) and concentrated elution fraction (lane 8).

Immobilised Metal Affinity Chromatography (IMAC) and Calmodulin affinity (CaM) was used to purify the recombinant γ-secretase enzyme complex. A range of detergents (including CHAPSO, Digitonin, Brij and DDM) at optimum concentrations have been tested to facilitate γ-secretase activity *in vivo*^60^. Endogenous γ-secretase retains maximal activity when extracted in zwitterionic detergent CHAPSO^60^, hence initial attempts at purifying γ-secretase were undertaken using CHAPSO. PS1(wt)-γ-secretase expressing insect cells membranes were prepared in 1% (w/v) CHAPSO and used as is or diluted to 0.5% or 0.25% CHAPSO to be applied to IMAC. Using these conditions, a substantial quantity of protein was detected in column flow-through and in subsequent washing steps, indicating poor binding of γ-secretase to the TALON resin (data not shown). Similar, results were obtained using Digitonin (used at 1%, 0.5% and 0.25%) and DDM (used at 0.05%) (data not shown). A combination of detergents was then investigated to improve binding of the recombinant γ-secretase to the TALON resin. Insect cell membranes were dissolved in 1% CHAPSO-Hepes buffer and diluted to 0.08% CHAPSO and 0.08% Digitonin (12.5 fold dilution). These detergents have been used at indicated concentrations to purify an intact and active γ-secretase complex^61^. Batch incubation of solubilized membranes with the TALON-IMAC resin was found to be more appropriate for maximal recovery of recombinant γ-secretase complex. Immunoblotting of purification fractions showed that the majority of the γ-secretase components were enriched in the same elution fractions (100 mM Imidazole; Fig. 5B–E, lanes 7, 8 and 9). Although a protein signal was observed in the diluted sample, flow-through and wash fractions, levels of NCT, PS1, APH1aL and PEN-2 (Fig. 5B–E; compare lanes 1–2 with lanes 8–9), were enriched in 100 mM Imidazole elution fraction and absent on the recovered TALON resin (data not shown) indicating that the bound components were almost completely eluted. We detected γ-secretase activity and thereby generation of AICD from APP-C99 Flag-tagged substrate only in the 100 mM Imidazole fractions (Fig. 5 lanes 7, 8 and 9). Although, γ-secretase components were detected by western immunoblotting in applied diluted sample, flowthrough and wash fractions (Fig. 5B–E; lanes 1–3), we did not detect generation of AICD in these fractions. We believe the concentration of recombinant γ-secretase complex in these fractions is lower than that required to produce AICD detectible by western immunoblotting.

The 100 mM Imidazole eluted IMAC fractions were pooled and batch incubated with Calmodulin resin (CaM). The resin was then washed and bound proteins were eluted with 10 mM EGTA and concentrated with a 50 kDa molecular weight cut-off centrifugal filter. Coomassie stain of SDS-PAGE gel indicated enrichment of γ-secretase components in the 50MWCO concentrated fraction (Fig. 5G, lane 8). Additionally, western immunoblotting for various γ-secretase components indicated the presence of γ-secretase components in the eluted 10 mM EGTA fractions (Fig. 5H–K, lanes 3–7) and were enriched in 50MWCO concentrated fraction (Fig. 5H–K, lane 8). Protein concentration of the 50MWCO concentrated fraction was measured using a BCA protein concentration estimation assay and indicated a yield of ~0.9 mg/litre (of cell culture) of purified recombinant γ-secretase enzyme complex.

Cell free assays established with IMAC and CaM affinity purification fractions indicated AICD generation in IMAC purified fractions (100 mM Imidazole, Fig. 5F lanes 6–8). Additionally, pooled IMAC fractions supplemented with CaCl_2_ and MgOAc (Fig. 5L, lane 1), CaM affinity flowthrough (Fig. 5L, lane 2, containing unbound recombinant γ-secretase components) and 50MWCO concentrated 10 mM EGTA elution fraction (Fig. 5L, lane 8) generated AICD from APP-C99 in cell free assays.

## Discussion

We have used the MultiBac baculoviral expression system to express and create an active γ-secretase enzyme complex, with activity comparable to γ-secretase from mammalian cells. Similar to previous studies in neuroblastoma and primary neuronal cells, overexpression of PS1 in HEK293/APPSwe cells led to an increase in Aβ42 production when compared to endogenous γ-secretase activity^62,63^. The activity of recombinant γ-secretase in generating AICD and NICD was specific to γ-secretase activity and could be blocked by a γ-secretase inhibitor in a dose dependant manner. Moreover, the activity of the reconstituted γ-secretase enzyme was retained following purification. Previous studies have utilised co-infection of multiple monocistronic baculoviral vectors to reconstitute γ-secretase in insect cells^25,27,45^. However due to differences in infectivity and protein production efficiencies of recombinant baculoviruses, it is often difficult and labour intensive to achieve a consistent relative equimolar expression form co-infected baculoviruses^64,65^. Importantly, co-transfection with multiple viruses reduces the efficiency of protein production and achieving stoichiometric expression of the reconstituted proteins is difficult^65–67^. In this study, we have circumvented these issues, by using the MultiBac multicistronic expression system to reconstitute an active γ-secretase enzyme complex. Using the techniques described above we obtained a yield of ~0.9 mg/L of purified recombinant γ-secretase complex. This yield is ~4.5 times greater than that observed in a previous study (0.2 mg/ml) employing a multicistronic mammalian vector^68^. The MultiBac has a number of features that allows for higher recombinant protein yields. The MultiBac employs a recombinant baculovirus DNA with disabled viral cathepsin and chitinase (cysteine protease) genes^30^ that are involved in viral mediated cellular liquefaction^32,33^, thereby improving protein production. Disabling these genes enables better compartmentalisation of recombinant protein production and reduces viral protease mediated degradation of recombinant proteins^30^. Additionally, the utility of MultiBac in studying γ-secretase is in its versatility to modular clone/exchange additional expression cassettes (the baculovirus can accommodate large heterologous DNA cargo)^69^. Hence MultiBac can be easily modified to facilitate investigation of potential protein modulators of γ-secretase activity^3,34–36^.

The complex glycosylation of MultiBac reconstituted Nicastrin is of particular interest. The majority of the Nicastrin expressed using this MultiBac baculoviral expression system is the highly glycosylated and at a molecular weight corresponding to that observed in mammalian cells^68,70^. This is in contrast to other baculoviral expression systems employing poly-cistronic baculoviral expression vectors^13,71^. This observations is encouraging considering proper maturation/glycosylation of Nicastrin is required for γ-secretase activity^49,72,73^. Similar to previous studies, the recombinant Nicastrin in our study is also resistant to complete deglycosylation by Endo-H^74^, indicating higher order glycosylation. It is possible that better compartmentalisation of MultiBac infected insect cell secretory pathway gives an opportunity for Nicastrin to fully process to its mature form. Previous studies have indicated that baculoviral infection leads to an upregulation of glycosylation enzymes and ER transport proteins in insect cells^75,76^. In addition, baculoviral infection also leads to an increase in COPII coated transport vesicles that are involved in shuttling transmembrane proteins from ER to the cis-Golgi^76^. However, the effect that the heterologous γ-secretase components expressed in this study have on intracellular transport needs to be considered. Indeed, PS1 is known to be involved in intracellular APP sorting^77^ and is found in association with COPI and COPII vesicles^78,79^. NCT interacts with ER retrieval signal Rer1p^80^. Although, the Sf9 genome is still rudimentary^81,82^, a role of Sf9 endogenous proteins transiently associating with γ-secretase and influencing intracellular transport/localisation cannot be discounted. Partial susceptibility to deglycosylation by EndoH enzyme indicate that insect cell expressed Nicastrin is at least rich in high-mannose glycosylation events. Nevertheless, mature mammalian Nicastrin is O-GlcNAcylated and sialylated^83^. The intrinsic limitation of the baculoviral/insect cell expression systems (and MultiBac) is in their inability to faithfully recapitulate post translational protein modifications of complex glycosylation and sialylation observed in mammalian proteins^84^. However, emerging baculoviral expression technologies now allow for expression of more mammalianised recombinant glycoproteins^85–89^. Further, the MultiBac expression system has been also optimised to produce complex N-linked glycosylated and galactosylated recombinant proteins^86^. Considering, the Nicastrin ectodomain functions to sterically hinder full length substrates^90^ from interacting with γ-secretase and glycosylation is involved in substrate selection^91^, it would be interesting to see if differential post translational modifications of Nicastrin observed in our system will allow substrates with longer ectodomain to interact with γ-secretase.

We have demonstrated the utility for the versatile MultiBac as an improved approach to express and reconstitute the γ-secretase enzyme. A recent study used a tetra-cistronic baculoviral expression vector to reconstitute and purify γ-secretase from insect cells^13^. While the yield of purified recombinant γ-secretase was not reported, Nicastrin was detected at a lower molecular weight (<98 kDa) than that detected in this study and previous studies where γ-secretase was overexpressed in mammalian cells^68^. Simpler Nicastrin glycosylation could further impact expression of Nicastrin especially due to high susceptibility of nascent Nicastrin to degradation^74^. Additionally, only the mature complex glycosylated Nicastrin is associated with γ-secretase complex and is essential to reconstitute activity^92^.

The MultiBac expression system was also used to express the PS1(Δ9) clinical mutant in the absence of the other γ-secretase components. Intriguingly, and consistent with previous reports^26,44,45^, the mutant PS1 was found to be active, albeit the activity was lower than that observed for PS1(wt)-γ-secretase. It has been suggested that the PS1(Δ9) is constitutively active due to deletion of an “autoinhibitory domain” that blocks the catalytic site of Presenilins^93,94^ or, as Ahn *et al*.^44^ suggested, appropriate positioning of the catalytic aspartic dyad. Nevertheless, baculovirally expressed PS1(Δ9) led to negligible amounts of Aβ42 production when compared to Aβ40 production, consistent with previous observations^45^. However, this finding is in contrast to activities of PS1(Δ9) in processing APP-C99 obtained by Ahn *et al*.^44^ and studies involving PS1(Δ9) expression with other γ-secretase components^57,58^. The reasons for this discrepancy are not clear. It has been suggested that γ-secretase substrates are first recruited by Nicastrin^95,96^ and interact with an exosite consisting of Nicastrin and PEN-2^97^. The exosite is thought to interrogate and recruit appropriate substrates^97^. It could be speculated that due to missing γ-secretase component involved in substrate recruitment, our PS1(Δ9) construct although constitutively active is also less efficient. Cell free assay reactions are inherently disordered and spatial restriction of enzyme on proteoliposomes^44^ or active recruitment of substrates^95,97^ would enhance enzymatic activity. However, we cannot discount the possibility that PS1(Δ9), produces Aβ species other than Aβ40 and Aβ42.

Overall, our findings have validated the utility of the MultiBac expression system to concertedly express components of heterologous protein complexes, such as γ-secretase. This baculoviral expression system can now be used as a method to further understand the functions of this important Aβ generating enzyme complex. In addition, the modular assembly employed in MultiBac would be particularly beneficial in understanding the effect of γ-secretase component heterogeneity on substrate specificity and the potential roles of protein modulators of γ-secretase activity^3,34–36^.

The system can also be adapted to characterize ADAD linked PS mutations and their effects on substrate binding and cleavage, thereby contributing to resolving the dominant gain or loss of function debate^98^. The γ-secretase cleavage of APLP1, APLP2 and APP, or fragments thereof, could be compared. Chemical inhibitor/modulator screening, selectivity and binding sites could be explored using the MultiBac system. MultiBac has also now been modified to expedite highly efficient transduction of large heterologous DNA cargo in more relevant *in vitro* models such as primary neurons and *in vivo* studies in zebrafish embryos (MultiPrime^69^). With high throughput pipeline for screening co-expression constructs currently available^99^, this exciting approach provides a new tool for the study of γ-secretase that can be manipulated in many ways to provide significant insight into γ-secretase structure and activity.

## Material and Methods

The MultiBac utilizes restriction enzyme digestion/ligation and ‘Cre-loxP’ site-specific recombination techniques (pFBDM, pUCDM and pSpl) making it more versatile. The pFBDM plasmid has two expression cassettes each containing multiple cloning sites (MCS 1 and MCS 2 for restriction enzyme cloning) and driven by promoters of baculoviral polH and p10 genes. Additionally, pFBDM backbone contains a multiplication module (containing unique restriction sites) and a LoxP site (for Cre-lox cloning) that facilitate modular insertion and exchange of expression cassettes from other vectors^38–40,67^. The EMBacY bacmid (contained within competent *E. coli* DH10EMBacY) has been engineered with *viral cathepsin* and *chitinase* genes disabled and a yellow fluorescent protein (YFP) expression cassette (under baculoviral promoter of ‘polH’ gene) allowing real-time monitoring of heterologous protein production. The EMBacY baculovirus DNA contains a mini-AttTn7 acceptor site to facilitate integration of the pFBDM plasmid (containing Tn7-R and Tn7-L donor sequences), while concomitantly allowing blue-white selection for detection of successful transposition^30,38^.

The cloning strategy for incorporating the γ-secretase components into pFBDM is shown in Fig. 1. Briefly, *PS1* and *APH1aL* or PS1(Δ9) were cloned directly into the pFBDM- expression cassettes within multiple cloning sites- 1 and 2 (MCS1 and MCS2) respectively. *PEN-2* expression cassette was modularly cloned using restriction digestion at the multiplication module (M) of pFBDM and *NCT* expression cassette was modularly cloned into pFBDM using Cre-Lox recombination to arrive at a single transfer vector referred to as pFBDM- γ-secretase containing cDNAs of the four components of γ-secretase enzyme complex.

### Construction of γ-secretase component expression cassettes

The PS1(wt), PS1Δ9, APH1aL, NCT and PEN-2 inserts were generated from human cDNA templates by polymerase chain reaction (PCR). PCR primers were designed to incorporate unique restriction endonuclease sites (Supplementary Table 1) to facilitate cloning in the subsequent steps. In addition, eight histidine repeats (octa-His tag) were cloned at the C-terminus of NCT and Calmodulin Binding Protein (CBP) purification tag was cloned at the N-terminus of PEN-2 to facilitate protein purification. The PCR amplified PS1(wt), PS1Δ9 and APH1aL inserts were cloned into pGEM^®^T easy (Promega A1360) vectors using ‘T-A’ cloning and maintained in *E. coli* One Shot^®^ MAX Efficiency^®^ DH5α™-T1^R^ (Life Technologies, Australia 12297016).

The NCT-octa-His insert was cloned into pSPL vector (pSPL vector described previously^38^). The pSPL vector contains a LoxP site and baculoviral promoters of ‘p10’ and ‘polH’ genes. The p10 promoter was removed using PmeI and BstZ171 restriction enzymes to generate pSPL-polH vector. This linearized vector was ligated with NCT-octa-His PCR product to generate vector pSPLpolh-NCT-octa-His, which was maintained in One Shot^TM^ PIR1 *E. coli* (Life Technologies, Australia C101010).

The PEN-2 insert was cloned into a modified version of previously described pUCDM vector^38^. This modified pUCDM vector contained a Calmodulin Binding Protein (CBP) and Strep Tag II protein purification tags followed by an enterokinase protease recognition sequence at the N-terminus of MCS. PEN-2 cDNA was restriction cloned at NdeI and RsrII restriction sites at the MCS to generate the pUCDM-PEN-2 vector, which was maintained in One Shot^TM^ PIR1 *E. coli*.

### Generation of recombinant pFBDM plasmids

The PS1(wt) and PS1(Δ9) inserts were excised from their respective pGEM^®^T vectors and cloned independently into the MCS1 of pFBDM plasmid using BamHI and HinDIII restriction endonucleases. This generated pFBDM-PS1(wt) and pFBDM-PS1(Δ9) respectively.

Similarly, APH1aL insert was excised from the vector pGEMT-APH1aL and cloned directly at XhoI and KpnI restriction endonucleases sites within the MCS2 of the pFBDM-PS1(wt) vector resulting in pFBDM-PS1(wt)-APH1aL vector. pUCDM- PEN-2 expression cassette was cloned into the above vector at the SpeI restriction site within the multiplication module (M) of the pFBDM plasmid, to generate pFBDM-PS1(wt)-APH1aL-CBP*-*PEN-2. Finally, pSPLpolH-NCT-octa-his was integrated into the pFBDM-PS1(wt)-APH1aL-CBP-PEN-2 vector using ‘Cre-Lox’ recombination to generate ‘pFBDM-PS1(wt)-γ-secretase’. All pFBDM derived plasmids were maintained in DH5α *E. coli*.

### Generation of recombinant MultiBac Baculovirus DNA

The recombinant pFBDM plasmids (pFBDM-PS1(wt)-γ-secretase and pFBDM-PS1(Δ9)) generated above were transformed into DH10EMBacY competent *E. coli*, containing the EMBacY bacmid^100^. The recombinant pFBDM plasmid transposes into the baculovirus DNA at the mini-attTn7 site, located within a *LacZ* gene, thereby disabling it and thus allowing for blue-white identification of successfully transposed colonies in presence of IPTG and BluoGal^38^. The colonies were selected in presence of gentamycin and kanamycin. This process generated two recombinant EMBacY bacmid containing either PS1(wt)-γ-secretase or PS1Δ9 cDNAs.

### Cell Culture and Generation of initial baculovirus

Human embryonic kidney cells over expressing ‘Swedish’ mutant of APP (HEK293/APPSwe cells) described before^101,102^ and human neuroblastoma SHSY5Y cells (ATCC, CRL2266) were cultured in DMEM (Sigma-Aldrich, UK D6546) and DMEM/F12 (Sima-Aldrich, UK D6421) respectively, supplemented with 10% FBS (Interpath, France SFBSF,) at 37 °C in 5% CO_2_. Insect Sf9 cells (Invitrogen; USA 11496-015) were maintained at 27 °C in ambient air as either adherent or suspension cultures (shaking at 120 rpm) in Sf900^™^-II-serum free media (Invitrogen; USA 10902-096). The recombinant or control (empty) EmBacY baculoviral DNAs were transfected into adherent cultures of Sf9 cells (1 × 10^6^ cells) using FuGENE^®^HD Transfection Reagent (Promega), using the manufacturer’s protocol. Initiation of YFP expression was taken as an indication of recombinant protein expression. The cells were monitored every 24 hours by bright-field microscopy to observe cell membrane disruption, indicating initial stages of viral mediated cell lysis. Conditioned media containing initial baculoviral particles (V0) was collected at <50% cell viability and used to infect fresh 10 ml Sf9 (1 × 10^6^ cells/ml) suspension culture to generate V1. The V1 baculovirus was used to infect 50 ml of Sf9 culture to generate baculovirus V2. The baculovirus V2 was saved in 5 ml aliquots at 4 °C for short term or −80 °C long term and used to infect larger Sf9 cultures. The V2 viral titre was determined using the baculoQUANT titration kit (Oxford Expression Technologies, UK), in accordance with the manufacturer’s protocol. Briefly, recombinant baculovirus DNA was extracted from viral particles and baculoviral gene gp64 was measured using qPCR and viral titre was estimated by plotting against a standard curve. Recombinant baculoviruses were pelleted from 80 µl of conditioned media by centrifuging at 16,000 × g for 5 minutes at room temperature. Next, the recombinant and internal standard baculoviruses (supplied) were resuspended in 20 µl of lysis buffer (supplied) and lysed in a thermocycler and heating to 65 °C for 15 minutes, 96 °C for 2 minutes, 65 °C for 4 minutes, 96 °C for 1 minutes, 65 °C for 1 minutes, 96 °C for 30 seconds. Next, 2 µl of recombinant baculovirus DNA was mixed with qPCR reagent (12.5 µl) and baculoviral gp64 gene primers and probes (3 µl) (supplied) to a final volume of 25 µl. The qPCR reactions were carried out in triplicates for each sample in a ViiA7 Real-Time PCR System (Applied Biosystems). Mean Ct values of unknown samples were plotted against the standard curve to obtain plaque forming unit (PFU)/ml of conditioned media.

### Protein expression analysis

Suspension Sf9 culture (10 ml at 1 × 10^6^ cells/ml) were infected with V2 at multiplicity of infection (MOI) of 1 PFU/cell. Every 12 hours post transfection; an aliquot of cell culture was harvested and cells were counted. Cell viability was estimated using 0.4% Trypan Blue exclusion method. Additionally, YFP expression was measured to estimate extent of baculoviral infection and recombinant protein expression. Every 12 hours 1 × 10^5^ infected insect cells were harvested, washed and resuspended in 200 µl of PBS. Following brief sonication (30% amplitude for 10 seconds on ice), cell debris was removed by centrifugation at 18,000 x g for 5 minutes at 4 °C. The supernatant was transferred to a 96 well plate and YFP was measured against a PBS blank in a fluorescence plate reader (excitation: 488 nm; emission: 520 nm).

Recombinant protein expression was observed by western immunoblotting of infected insect cell lysates. Cell lysates were prepared by homogenising cells in Nonidet P-40 cell lysis buffer (50 mM Tris-HCl, 150 mM NaCl, cOmplete^TM^ protease inhibitor cocktail (Roche, Germany), 2 mM EDTA and 1% Nonidet P-40 detergent v/v) and incubating on ice, with intermittent vortexing every ten minutes, for a total time of 30 minutes. Soluble proteins were extracted by centrifuging the homogenised cells at 18,000 × g at 4 °C for 10 minutes. Protein concentration was estimated using BCA assay (Micro BCA Protein Assay Kit, Thermo Scientific USA 23235). Approximately 5 µg of total proteins were resolved on 4–12% SDS-PAGE gels and γ-secretase components were detected by Western immunoblotting^45^. Well characterised primary antibodies were used; NT1 (1:5000, PS1 N-terminal, kindly provided by Professor Paul Fraser University of Toronto^103^); Ab14 (1:5000, PS1 N-terminal, kindly provided by Professor Sam Gandy Mt Saini Hospital, NY^103^), Nicastrin (1:1000, Sigma-Aldrich, USA N1660); PEN-2 (1:1000, Sigma–Aldrich, USA P5622) and APH1aL (1:1000, Sigma–Aldrich, USA A9603). Other primary antibodies used in this study were WO2 (1:5000, APP/Aβ, kindly provided by Professor Colin Masters University of Melbourne); 6E10 (1:1000, APP/Aβ, Covance USA 39300), penta-histidine antibody (1:1000, Qiagen, Germany 34660) and Flag-M2-HRP (1:1000, Sigma–Aldrich, USA A8592). Immunoblots were developed using electrochemiluminescence reagent (Amersham ECL, GE, USA RPN2232; or BioRad, USA 1705061) and protein bands were detected using X-ray films (Amersham Hyperfilm ECL, GE, USA 28906835) or ChemiDoc XRS^+^ (BioRad).

### Deglycosylation experiments

Human neuroblastoma SHSY5Y cells or PS1(wt)-γ-secretase infected Sf9 cell lysates were prepared in Nonidet P-40 lysis buffer as mentioned above. Next, 5 µg of PS1(wt)-γ-secretase expressing insect cell lysate and 50 µg of SHSY5Y cell lysates were denatured and deglycosylated with either Endo-H (NEB P0702) or PNGase -F (NEB P0704) enzymes following manufacturer’s protocol.

### Co- Immunoprecipitation studies

Co-Immunoprecipitation was carried out as described previously^104,105^. Infected insect cell membranes were dissolved in 1% CHAPSO-HEPES buffer (25 mM HEPES, pH7.4; 150 mM NaCl; 1% CHAPSO w/v; Buffer A) and diluted to 1 mg/ml protein concentration, using the same buffer. Soluble membranes were incubated with PS1 antibody Ab14 (rabbit polyclonal raised against 1–25 amino acids of PS1) overnight at 4 °C under gentle agitation. Protein-A Mag Sepharose (GE, UK 28951378) was used to harvest protein antibody complexes and eluted in LDS sample buffer (Invitrogen, USA NP0007) at 37 °C for 15 minutes for SDS-PAGE and Western immunoblotting.

### Preparation of cell membranes from Sf9 cells

Sf9 suspension cultures were infected with recombinant baculoviruses at MOI of 1 PFU. Following maximal protein expression (as monitored by YFP measurement), cells were collected by centrifugation at 300 × g for 5 minutes at room temperature. The cell pellet was homogenised in equal volume of homogenising buffer (5 mM Hepes pH 7.4; 250 mM Sucrose; 1 mM EDTA and cOmplete^TM^ protease inhibitor) and lysed by drawing in and out of a 26 gauge needle, ~15 times (for small 25–50 ml cultures) or freeze thawing cell suspension (for 500–1000 ml cultures). Cell and nuclear debris were removed by centrifugation at 800 × g for 10 minutes at 4 °C. The supernatant was transferred to ultracentrifugation tubes and the cell membranes were pelleted by centrifugation at 100,000 × g for 60 minutes at 4 °C. The membrane pellet was stored at −80 °C until required. The membrane pellet was resuspended in buffer A and homogenised using a 26-gauge needle, drawing in and out 15 times (for small 25–50 ml cultures) or 20 strokes of a Dounce homogeniser (for 500–1000 ml cultures) on ice. The cell membranes were allowed to solubilise at 4 °C for 1 hour, using end over end rotation (10 rpm). Insoluble membranes were removed by centrifugation at 100,000 × g for 60 minutes at 4 °C and the supernatant containing dissolved membranes was used immediately.

### Purification of reconstituted γ-secretase enzyme complex

PS1(wt)-γ-secretase infected Sf9 cell membranes (100 ml culture growing at 2 × 10^6^ cells/ml) were prepared as mentioned above in 1% CHAPSO- HEPES buffer (Buffer A, containing cOmplete™, EDTA-free Protease Inhibitor Roche 11873580001; henceforth called buffer B). The membranes were then diluted to 0.08% CHAPSO and 0.08% Digitonin (from a 2% stock solution) using Buffer B. The sample was then incubated at 4 °C with end to end rotation of 10 rpm for 16 hours with 3 ml of His TALON, IMAC resin (GE, Sweden 28957502) pre-equilibrated with 0.08% CHAPSO and 0.08% Digitonin- Buffer B. The resin was collected into an empty PD10 column (GE, USA 17043501) and flowthrough was saved. The resin was then rinsed with 30 ml of buffer B to remove unbound proteins. Next, the resin was rinsed twice in 3 ml of 5 mM Imidazole in Buffer B, to remove any weekly bound proteins. Finally, γ-secretase was eluted in 3 ml fractions of 100 mM Imidazole in Buffer B.

Next, IMAC purified 100 mM Imidazole fractions were pooled and supplemented with 2 mM Calcium Chloride (CaCl_2_) and 2 mM Magnesium acetate (MgOAc). The pooled fractions were then incubated at 4 °C with end to end rotation of 10 rpm for 16 hours with 3 ml of Calmodulin affinity Resin (CaM) (GE, Sweden 17052901) equilibrated in buffer B supplemented with 2 mM CaCl_2_ and 2 mM Magnesium acetate (Buffer C). The resin was rinsed in 30 ml of Buffer C and eluted in 3 ml fractions of 0.5% CHAPSO in 25 mM Tris, 150 mM NaCl and 2 mM MgOAc (Buffer D) supplemented with 10 mM of EGTA. The 10 mM EGTA fractions were pooled and concentrated with a 50 kDa molecular weight cut-off centrifugal filter (50MWCO, GE USA 28932362). The IMAC and CaM purification fractions were resolved on a 4–20% SDS-PAGE and various components of γ-secretase enzyme complex were detected by Coomassie stain and Western immunoblotting.

### Cell free γ-secretase assay

*In vitro* cell-free assays were performed using *E. coli* expressed APP-C99-Flag (N-terminal 99 amino acids of APP-CTF, starting with an additional methionine and a Flag tag at C-terminus) and Notch-C100-Flag (N-terminal 99 amino acids of NotchΔE, starting with an additional methionine and a Flag tag at C-terminus) as previously described^54,55,106^. Briefly, APP-C99-Flag and Notch-C100-Flag inserts were cloned into pET29 vector and transformed into BL21(DE3) *E. coli* cells (New England Biolabs) for substrate expression. Recombinant protein expression was induced using 1 mM IPTG following the manufacturer’s protocol. The cells were collected by centrifugation at 8000 × g for 10 minutes at 4 °C. Cell pellet was re-suspended in solubilising buffer (50 mM Tris-HCl pH8.0, 150 mM NaCl and cOmplete^TM^ protease inhibitor). The cell suspension was sonicated 6 times in an ice bath at 15% amplitude for 10 seconds, with intermittent cooling of 1 minute each. The homogenate was then supplemented with 1% Nonidet P-40 (v/v) detergent and incubated on ice for 1 hour. Detergent soluble substrates were collected by centrifuging the homogenised cells at 20,000 × g for 10 minutes at 4 °C. The solubilised substrates were aliquoted and rapidly frozen in liquid nitrogen and stored at −80 °C.

Insect cell membranes dissolved in 1% CHAPSO-Hepes buffer were diluted to 0.5% CHAPSO with 150 mM Sodium citrate, (pH 6.4) and mixed with equal parts of cell free assay reaction buffer (150 mM Sodium Citrate (pH 6.4); 20 mM Dithiothreitol (DTT); 0.1% phosphatidylcholine (PC) lipid (Avanti Polar Lipids, USA 840053); 0.025% phosphatidylethanolamine (PE) lipid (Avanti Polar Lipids, USA 840022); 0.2% BSA; 2 mM EDTA; cOmplete^TM^ protease inhibitor cocktail (Roche, 500 mg/ml) and 10 µl of substrate). Final detergent concentrations were 0.25% CHAPSO and <0.05% Nonidet P-40. Enzymatic reactions were carried out at 37 °C, for 16 hours and terminated by adding LDS gel loading sample buffer/freezing at −20 °C. Similarly, IMAC and CaM purification fractions were incubated with equal volumes of reaction buffer containing appropriate substrate. The reaction products were resolved by SDS-PAGE and immunoblotted to detect AICD/Aβ or NICD. Levels of Aβ40 and Aβ42 peptides generated in three different cell free assay experiments were quantified using ELISA kits (Life Technologies, Austria KHB3441 and KHB3481), following manufacturer’s protocols.

## Electronic supplementary material

Additional Information
