# Supplementary material for: Efficient production of a mature and functional gamma secretase protease
Source: Sci Rep. 2018 Aug 27;8:12834. doi: 10.1038/s41598-018-30788-w (PMC6110731; doi:10.1038/s41598-018-30788-w)
Supplement: Supplementary file 1 — Additional Information [file 41598_2018_30788_MOESM1_ESM.docx]

# Efficient production of a mature and functional gamma secretase protease.

Imran Khan^1,2,3^*, Sudarsan Krishnaswamy^3^, Miheer Sabale^1^, David Groth^1^, Linda Wijaya^3^, Michael Morici^3^, Imre Berger^4,5^, Christiane Schaffitzel^4,5^, Paul E. Fraser^6^ and Ralph N. Martins ^2,3,7^ Giuseppe Verdile^1,2,3^*

**Supplementary information**

**
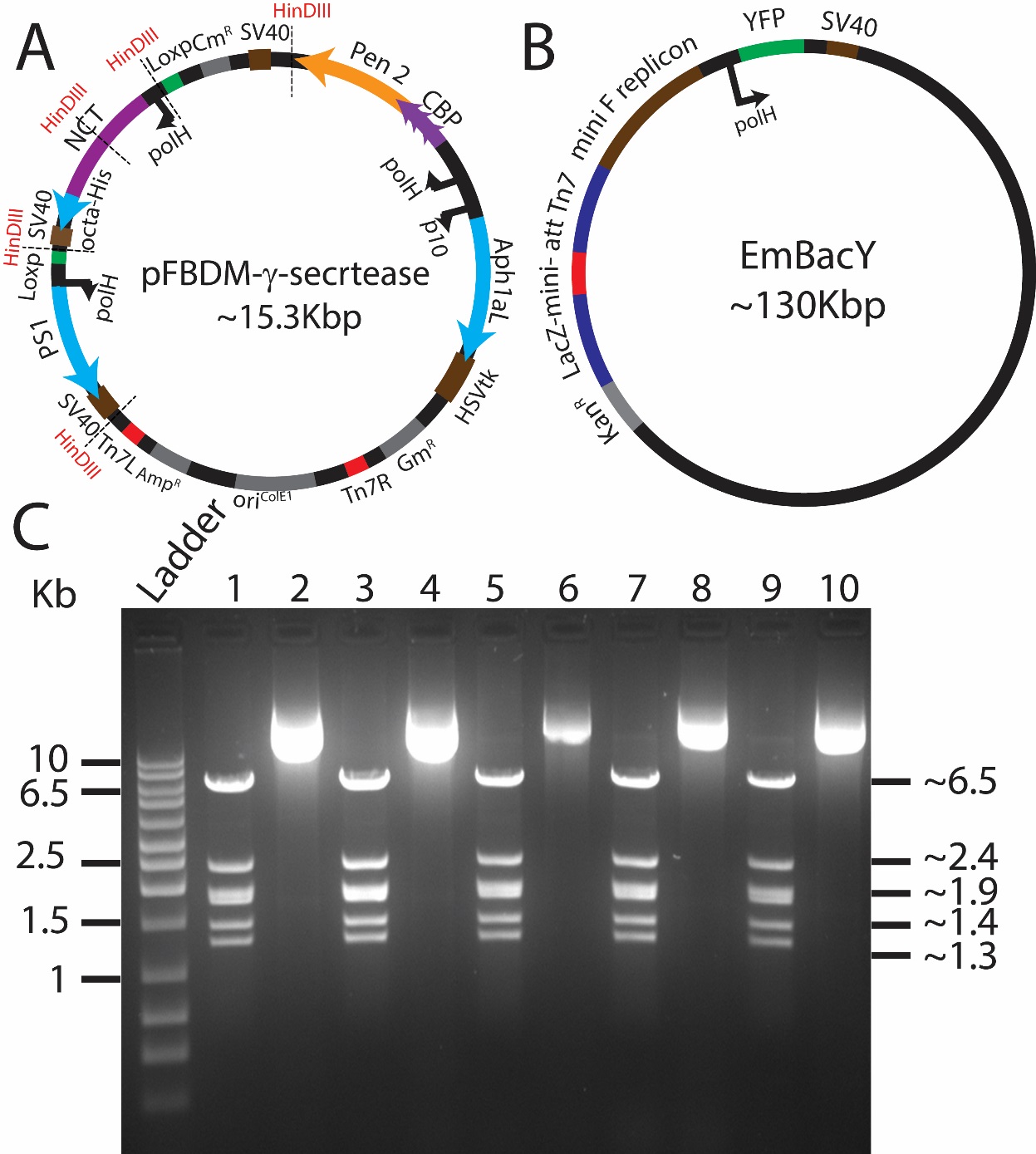
**

Figure S 1: Restriction digestion of EmBacY γ-secretase

Expression vector pFBDM-γ-secretase (PS1wt) was miniprep form DH5α *E.coli* and 1µg of DNA was digested with HinDIII restriction enzyme. The reaction products were resolved on a 0.8% agarose gel. (A) Map of pFBDM-γ-secretase (PS1wt) expression vector with HinDIII restriction sites indicated. (B) A map of the EmBacY indicating the bacterial β-galactosidase gene (LacZ), mini attTn7 transposition site and YFP expression cassette (C) DNA from 5 individual colonies was digested with HinDIII (C lanes 1,3,5,7 and 9, digested*,* lanes 2, 4, 6, 8 and 10 corresponding undigested DNA) and resulted in generation of correct size fragments as indicated in (A).

Table 1: List of primers to amplify cDNAs from transfer vectors, containing the restriction enzyme sites (blue and underlined) required for cloning into pFBDM.

| **Primer** | **Sequence (5’ → 3’)** | **(bp)** |
| --- | --- | --- |
| PS1- F | AAAAAAAA**GGATCC**GCAATGACAGAGTTACCTGCAC CGTTGT | 42 |
|  | BamHI |  |
| PS1 -R | AAAAAAAA**AAGCTT**TTAGATATAAAATTGATGGAAT GCTAATTG | 44 |
|  | HinDIII |  |
| APH1aL- F | AAAAAAAA**CTCGAG**ATGGGGGCTGCGGTGTTTTTCG GC | 38 |
|  | XhoI |  |
| APH1aL- R | AAAAAAAA**GGTACC**TCAGTCCTCGGGTGGGATGC | 34 |
|  | KpnI |  |
| C100 -F | AAAAAAAA**CTCGAG**CAATGGATGCAGAATTCCGAC | 35 |
|  | XhoI |  |
| C100 -R | AAAAAAAA**GGTACC**CTAGTTCTGCATCTGC TCAAAGAAC | 39 |
|  | KpnI |  |
|  |  |  |
| NCT-F | GGAATTCC**AGATCT**ATGGCTACGGCAGGGGGTGGCTC TGGGGCTGACC | 48 |
|  | BglII |  |
| NCT-His-R | GGAATTCC**TCTAGA**TTAT**CA**GTGATGATGGTGATGATGGTGGTG | 90 |
|  | XbaI octa his tag |  |
|  | **ACCGCC**GTATGACACAGCTCCTGGCTCCCGGGGAGCAATGAAAAGG |  |
| PEN2-F | GGAATTAAC**CATATG**AACCTGGAGCGAGTGTCCAAT GAGGAG | 42 |
|  | NdeI |  |
| PEN2-R | TGGAATT**CGGACCG**TTA**TCA**GGGGGTGCCCAGGGGTATGGTGAAGGAG | 48 |
|  | RsrII |  |
